# Reducing bias in trials due to reactions to measurement: experts produced recommendations informed by evidence

**DOI:** 10.1016/j.jclinepi.2021.06.028

**Published:** 2021-07-03

**Authors:** David P French, Lisa M Miles, Diana Elbourne, Andrew Farmer, Martin Gulliford, Louise Locock, Stephen Sutton, Jim McCambridge

**Affiliations:** a Manchester Centre for Health Psychology, University of Manchester, Oxford Road, Manchester, UK; b Department of Medical Statistics, London School of Hygiene and Tropical Medicine, Keppel Street, London, UK; c Nuffield Department of Primary Care Health Sciences, University of Oxford, Oxford, UK; d School of Population Health and Environmental Sciences, King’s College London, London, UK; e Health Services Research Unit, University of Aberdeen, Aberdeen, UK; f Behavioural Science Group, Department of Public Health and Primary Care, University of Cambridge, Cambridge, UK; g Department of Health Sciences, University of York, York, UK

**Keywords:** Bias, Measurement reactions, Reactivity, Recommendations, Research design, Trials

## Abstract

**Objective:**

This study (MEasurement Reactions In Trials) aimed to produce recommendations on how best to minimize bias from measurement reactivity (MR) in randomized controlled trials of interventions to improve health.

**Study design and setting:**

The MERIT study consisted of: (1) an updated systematic review that examined whether measuring participants had effects on participants’ health-related behaviors, relative to no-measurement controls, and three rapid reviews to identify:(i) existing guidance on MR; (ii) existing systematic reviews of studies that have quantified the effects of measurement on behavioral or affective outcomes; and (iii) studies that have investigated the effects of objective measurements of behavior on health-related behavior; (2) a Delphi study to identify the scope of the recommendations; and (3) an expert workshop in October 2018 to discuss potential recommendations in groups.

**Results:**

Fourteen recommendations were produced by the expert group to: (1) identify whether bias is likely to be a problem for a trial; (2) decide whether to collect data about whether bias is likely to be a problem; (3) design trials to minimize the likelihood of this bias.

**Conclusion:**

These recommendations raise awareness of how and where taking measurements can produce bias in trials, and are thus helpful for trial design.

## 1 Introduction

Measuring people can affect the people being measured, producing changes in behavior, emotions and the data they provide about themselves [1–3]. This phenomenon is known as measurement reactivity (MR) [1]. Randomized controlled trials (RCTs) always include measurements of trial outcomes such as self-reports, objective measurements of behavior and/or clinical markers, and possibly further measurements as part of process evaluations. The usual methods of conduct and analysis of trials implicitly assume that measurements do not affect subsequent outcome measurements or interact with the trial intervention, and that any effects will be the same in each experimental group, and are therefore unlikely to bias treatment comparisons [1,2,4]. Possible effects on participants are ignored, and not considered as a potential source of bias.

The most compelling evidence of MR is found in two areas: (1) the question-behavior effect, where measurement in the form of asking questions about behavior, produces small changes in the behavior being asked about [3,5,6], and (2) the effects of measuring physical activity using pedometers (particularly where step counts can be read by participants) producing increases in physical activity [7,8]. In addition, there is also evidence from randomized studies showing that completing questionnaires about the consequences of illness results in people reporting higher anxiety levels than people who have not completed them [9]. Other measurement procedures widely employed in RCTs, such as assessing bodyweight, are also used as intervention techniques in their own right, as they are seen to be effective at producing behavior change [10]. In many areas of research the possibility of MR has been insufficiently considered or investigated [11]. As a consequence, MR is not adequately addressed within existing guidelines for designing, reporting and appraising trials.

The MERIT (MEasurement Reactions In Trials) study was funded by the MRC and NIHR [12] to produce recommendations on how and where taking measurements can lead to bias and to provide recommendations to minimize such bias. It involved updating a systematic review, conducting three rapid systematic reviews and a Delphi study, to generate an evidence base, which an expert workshop used to produce recommendations. The full methods for the MERIT study and the findings from the evidence reviews are published separately [6,12], a brief summary of which is presented in Box 1. The present report provides contextual background on how MR can lead to bias and a summary of the recommendations generated from the MERIT study.

## 2 Measurement reactivity and risk of bias

To inform recommendations about how to limit bias due to MR, we identified six plausible scenarios in which MR may produce bias. It is important to note that the existence of MR is not sufficient by itself to produce bias in a trial’s intervention effect estimate.

### 2.1 Different measurement protocols across trial arms

Bias may arise when different measurement protocols are used across randomized trial arms, with one trial arm being measured differently from another. If measurement impacts on trial outcomes, greater disparities in measurement protocols will produce greater bias. For example, participants in the experimental condition may complete the following measurements, whilst participants in the control condition are not asked to complete such measures: (1) process measures to assess intervention mechanisms of effect, (2) momentary assessments of behavior or treatment response (ie, repeated data collection in an individual’s usual environment, at or close to the time they carry out that behavior) using technology such as a digital application, or (3) intervention feedback or fidelity assessment (to determine whether the intervention is delivered as intended). These measures carry the potential to impact upon a participant’s experience of the intervention, negatively or positively. For example, the measures could serve as reinforcers, reminders or boosters of intervention effects, and thus can exaggerate the apparent effects of interventions.

### 2.2 Contamination

Contamination refers to the inadvertent exposure of a non-experimental control group to intervention content that is an integral part of an effective experimental group treatment. For instance, if a pedometer were one intervention component in a multi-component intervention to increase physical activity, then its use as a measure to evaluate change in physical activity in both arms is intrinsically problematic, as the non-intervention control group are gaining access to part of the intervention. Similarities between the contents of research measurements and interventions provide grounds for concern about bias being induced by contamination. In this situation, estimates of effectiveness are likely to be biased towards the null, as both intervention and control groups are exposed to similar content.

### 2.3 Interactions between measurement and intervention

Where research procedures mimic the effect of the intervention via similar mechanisms, this may produce bias, even when the research procedure is not part of the intervention. For example, self-monitoring may be promoted in a study by both a measurement procedure such as self-weighing and an intervention procedure such as regular use of a bodyweight diary. Thus, the use of self-weighing as a measurement procedure may interfere with comparisons between randomized groups, as both may be exposed to content that underpins the anticipated effect of the intervention. In this example, the biasing effect will be similar to that of contamination, that is, towards the null. In principle, bias could go in either direction: for example, research measurement could prepare experimental group participants to be more receptive to an intervention by prompting contemplation of the reasons for behavior change [4].

### 2.4 Ceiling effects

Restrictions on the possible range of measured out-comes can interact with MR where measurement impacts on both arms in a two-arm trial [4]. For example, there may be a finite limit to the amount of fruit and vegetables that a dietary intervention can reasonably stimulate. The more that measurement procedures unintentionally stimulate the behavior that is the target of the intervention, the less scope there is for the intervention to stimulate changes in diet further and hence be more effective than the control.

### 2.5 Measurement reactivity as a component of performance bias

Where MR is present, there are both clinical and research practices associated with measurement that can lead to bias, rather than the measurement *per se*. For example, the process of collecting measurement data during the course of a trial may alter the care provided by healthcare professionals (eg, more or less attention, ancillary treatment and diagnostic investigations), which may lead to bias if such alterations are implemented differently for randomized groups. For example, one group of patients may have more frequent contact with healthcare professionals as a result of regular assessment of bodyweight, blood pressure, or blood tests to assess liver function. This is a specific case of the wider class of performance bias.

### 2.6 Effects on attrition bias

Reactions to measurement can also be implicated in other forms of bias. For example, from the participant’s perspective, too much measurement can increase the burden of trial participation so that they decide to drop out. Where the burden differs between intervention and control group participants, MR may be more likely to produce differential attrition.

## 3 Recommendations

Recommendations produced by the expert workshop were grouped into three broad types: first, identifying whether bias is likely to be a problem for any specific trial; second, deciding whether to collect further data to inform decisions about whether bias is likely to be a problem; and third, how to design trials to minimize the likelihood of this form of bias.

### 3.1 Identify whether measurement reactivity is likely to be a major source of bias for a trial

In many circumstances, whilst bias from MR may be present, it is often likely to be of small magnitude [6] compared to other potential sources of bias, such as failure of randomization [13], and so can safely be ignored. However, it is worth considering that reactions to assessment can exacerbate or contribute to other sources of bias that are already well-recognized [2].

#### Recommendation 1. *Consider potential for measurement reactivity causing bias at the outset of designing a trial*

It will be easier to prevent MR causing bias than to deal with the consequences of bias through analysis after the event. Given this, researchers should consider at the out-set whether the trial they are planning is likely to produce bias. It is important to consider the many measurement and assessment processes involved in a trial, including assessment of eligibility, baseline assessments, assessments of adherence, assessments of fidelity, process evaluations (quantitative and qualitative), and interim and final outcome assessments. It is necessary to be clear where measurements are integral to an intervention (ie, as would be rolled out in practice), and to make decisions about research measurements in light of this knowledge.

#### Recommendation 2. *Consider potential for measurement reactivity as a source of bias at all stages of the research process*

It is important to consider all instances of measurement throughout the research process and how participants may react. For instance, when assessing eligibility of a potential trial participant, disclosure of health status (eg, blood pressure or cholesterol level) could make participants more or less receptive to an intervention [14]. Baseline measurements in a trial typically contribute to efficient design by enabling more precise estimation of the intervention effect. However, experiences of earlier measurement may influence responses at later measurement occasions and/or interact with the study intervention [11]. The prospect of future measurement may also produce changes in research participants. Further, electronic monitoring of medication adherence can lead to changes in adherence [15]. That is, knowledge or anticipation of measurement or disclosure of outcomes should be considered as potential sources of reactivity, as well as actual measurements conducted. Consider also people delivering interventions, who may exhibit greater adherence to intervention protocols when their delivery is recorded in a trial than would otherwise be the case. Standard Operating Procedures for measurement procedures should be consistent across trial arms, reduce unnecessary measurement occasions etc., and also address informal contacts/communications regarding assessment between trial participants and health care providers or trial personnel.

#### Recommendation 3. *Consider specific trial features that may indicate heightened risk of bias due to measurement reactivity* (Table 1)

Table 1 provides a series of trial features that might indicate where MR is a possible risk of bias in a study, based on the views of experts informed by evidence reviews [12]. The entries in Table 1, or “red flags” features of study design, indicate potential for bias from MR. This may prove absent on closer examination, or identified but mitigated through careful study design. The potential for measurement as a co-intervention leading to bias is not widely articulated in existing tools intended to assist study design.

#### Recommendation 4. *Theorize potential measurement reactions as part of a logic model of how an intervention is intended to work*

It is good practice to construct a logic model that specifies how an intervention results in the intended outcomes and helps in selecting appropriate measures and makes theory explicit in a trial [16]. It has also been proposed to develop models of “dark logic” by which interventions may produce unintended harmful effects, to better understand such phenomena [17]. In line with this proposal, researchers may explicitly theorize to what extent the risk of bias scenarios above may be applicable to their trial. This could involve potential research participants, as well as drawing on the knowledge of the research team.

#### Recommendation 5. *Consider the potential impact of measurement procedures on participants in comparison with the intensity and duration of the studied intervention*

While regular contact with research personnel may help sustain participants’ engagement and thereby continuation in a trial, it may be preferable to use non-measurement related activities (such as newsletters etc.) to support participants’ engagement. Circumstances in which the amount of contact or interaction with researchers or clinicians for baseline and follow-up measures are greater than intervention exposure are of particular concern, for example, in evaluations of brief interventions.

#### Recommendation 6. *Consider how participants may use measurement to meet their own aims*

It may be helpful to consider participants as motivated or rationally pursuing personal goals when considering the possible effects of MR in producing bias. This means paying careful attention to how participants engage with the features of trial design. For instance, people may wish to take part in a trial to gain access to outcome measurements such as blood pressure or to receive regular feedback on their activity levels from an accelerometer.

Where participants are being assessed by healthcare professionals with whom they have regular contact, they may wish to respond in such a way as to create a particular impression of need (to elicit services), or competence (if they wish to create a productive relationship). Thus, regular measurement may produce changes in selfreported outcomes. In the absence of blinding, participants in trials may exaggerate the personal benefits of a treatment, if they believe that the treatment should be more widely available. Arguably, the more extensive or meaningful the measurement is to the participant within a trial, for example, when involving additional checks for people with diabetes, or regular monitoring for relapse in people who have had cancer, the more likely that participants may use measurements to meet their own aims.

### 3.2 Collect further data to inform decisions about risk of bias resulting from measurement reactivity

Given the limitations of existing knowledge, researchers will sometimes have reason to be concerned that MR may be a problem for their trial, but find it challenging to assess risk of bias. In such circumstances, it may be sensible to collect further quantitative or qualitative information to inform decisions about potential modifications to trial design.

#### Recommendation 7. *Consider whether MR concerns for your trial warrant further empirical examination*

**Fig. 1:**
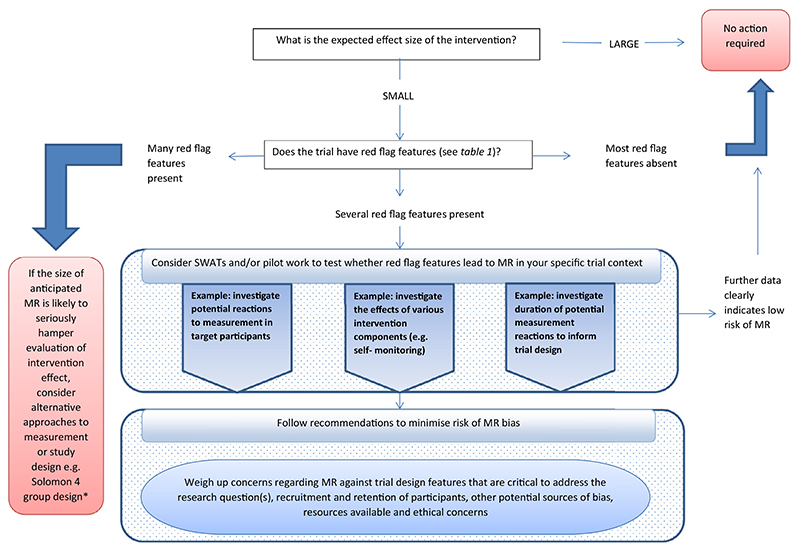
Flowchart to support decision making for recommendation 7. *Solomon 4 group design: a factorial design trial in which participants are randomized to intervention and control trial arms, as well as to baseline assessment or no baseline assessment

Having gone through processes indicated above, a judgement is required about the likelihood of risk of bias resulting from MR for a particular trial, and whether any further action is needed. Fig. 1 shows a flowchart to support decision making with options ranging from taking no further action and proceeding with the trial as planned, to further investigation.

Action taken needs to be proportionate and weighed against other priorities and concerns. In some situations, it may be appropriate to investigate likelihood of MR in feasibility studies. Qualitative studies could be informative in terms of understanding how people could potentially react to measurements. A further possibility is the incorporation of nested methodological studies (studies within a trial, SWATs) to estimate the magnitude of bias from MR in a subset of participants. The size of a SWAT is necessarily constrained by the size of the host trial and so results may be imprecise. A SWAT can nevertheless contribute to the overall body of evidence if results are combined in meta-analyses. Intensive approaches such as a Solomon four-group design are warranted only in trials where several indicators suggest that MR is a major concern and likely to bias effect estimation. Solomon designs involve a factorial design in which participants are randomized to intervention and control trial arms, as well as to baseline assessment or no baseline assessment [11]. An alternative approach is to consider a large simple trial that eschews baseline measurement altogether, thus relying on randomization of large numbers to generate equivalence between arms to safeguard the experimental design.

#### Recommendation 8. *Examine feedback from research personnel regarding research participants’ reports of changes in their behavior/thoughts/emotions as a result of measurement*

During the course of a trial, research personnel may often have several informal conversations with participants. It is possible that research participants might volunteer information about changes in behavior, thoughts or attitudes that have arisen from their participation in the trial, or specifically from measurement procedures. Including provision for the gathering of such material may be worth considering prior to seeking ethical approval. Where research personnel consistently provide feedback regarding the presence of MR, this could inform further process evaluations, statistical analysis strategies and/or interpretation of study findings.

### 3.3 Potential actions to minimize risk of bias from measurement reactivity within a trial

Where consideration of the issues suggests that MR is likely to cause bias within a trial, a number of options are available.

#### Recommendation 9. *Consider possible measurement reactivity when determining the overall burden of measurement in a trial*

Many patient-reported outcomes are collected during research, but often not analyzed or reported [18]. This has many downsides, including being an unethical use of participant time (especially when they are in poor health), respondent fatigue and lower response rates leading to poorer quality data. These are arguments for reducing measurement in trials. In addition, where measurements may induce reactivity, having less measurement may limit the scope for bias.

Measures of intervention process help researchers understand how and when effects are produced [16]. However, researchers may wish to consider when it is appropriate for all participants to complete all measures. Process measures typically assess hypothesized determinants of behaviors, such as attitudes or intentions. There is good evidence that asking people to complete these kinds of measures can affect behavior [3]. Asking all research participants to complete process measures will almost certainly be unnecessary as, if the hypothesized mechanism of action is correct, the effect size will be considerably larger than will be the case for the main outcome measure. It is reasonable therefore to ask only a subset of participants to complete process measures. Similarly, it may also be efficient to investigate two or more hypothesized causal pathways with randomly drawn, or targeted, sub-samples of participants in a single trial, with the same primary outcome measure.

#### Recommendation 10. *Embed measurement procedures into routine clinical practice where possible*

The use of unobtrusive measures has long been recommended to avoid problems of measurement affecting participants in research [19]. It will often be desirable to use measurements that are not collected primarily for research purposes, for example data in routine health records or existing data collected for other purposes to minimize the threat of bias from MR.

#### Recommendation 11. *Use identical measurement protocols in all arms of a trial*

In line with established good practice for trial design, it is desirable to ensure that measurement procedures are identical across all arms of a trial. This involves ensuring that all measurements are completed in the same setting at the same frequency and time-points, and where relevant, by the same types of people (eg, research nurses, GPs). Format and methods should also be identical; for example, online/pencil and paper questionnaire, semi-structured interviews.

Sometimes differential measurement procedures across arms of a trial are employed to help address the research question, for example, to monitor physiological effects in the intervention group only. Researchers in these types of studies should consider the implications carefully. In general, having balanced measurement across conditions introduces fewer problems than having unbalanced measurement, although having as little measurement as possible is usually the least problematic.

#### Recommendation 12. *Avoid overlap between measurement and intervention*

Some measurement techniques are similar, if not identical, to intervention techniques designed to change health-related behavior. For example, as noted earlier, pedometers are an efficient method of allowing people to self-monitor their behavior, when the users are not blinded to outcome. Use of pedometers leading to self-monitoring is a specific case of a general issue: it is a clear threat to the validity of a trial if measurement techniques are used that closely resemble one or more behavior change techniques that the trial is designed to evaluate. To inform theorizing about how the measurements may constitute active interventions, a standardized taxonomy has distinguished 93 techniques for changing behavior [20]. Measurement can mimic several of these techniques, by for example, serving as a prompt, promoting monitoring behavior, outcomes or emotional reactions, providing feedback on behavior or physiological indices, altering attention to previous successes or failures, and providing information about health threats.

#### Recommendation 13. *Consider the potential benefits of masking measures and/or withholding feedback of measured values*

Withholding information about which health-related measures are being collected, or the purposes or values of such measurements, could sometimes help reduce the risk of MR producing bias. In certain circumstances the aims of the research may be compromised by giving full information prior to data collection; this is particularly pertinent when there is potential for MR. For example, there is evidence that covert sealed pedometers (described as “posture monitors” to participants) do not lead to MR (increased physical activity) compared to use of an unsealed pedometer [8].

Information given to potential research participants, however, needs to be comprehensive to facilitate fully informed consent. It is therefore imperative that ethical considerations are taken into account before making decisions on masking of measurements are made. Where an essential element of the research design would be compromised by full disclosure to participants, the withholding of information should be specified and appropriately justified in the trial protocol and ethical approval submission. It is crucial that the research objective has strong scientific merit and that an appropriate risk management and harm alleviation strategy is in place. Further, the amount of information withheld and the delay in disclosing the withheld information should be kept to the absolute minimum.

#### Recommendation 14. *If measurement reactivity is likely to be present, investigations for measurement reactivity should be included a priori in the statistical analysis plan*

If there is some reasonable likelihood of MR being present, quantitative investigations of MR should be included *a priori* in the trial protocol and statistical analysis plan. These investigations could include sensitivity analyses based on, for example, a subgroup of trial participants measured more intensively in a sub-study in both trial arms.

Statistical analyses should also be informed by feasibility and pilot work (see recommendation 7). For example, for some measurement procedures such as blood pressure, self-reported anxiety or step count using pedometers, the first one or two measurements are particularly reactive. For this reason, some researchers collect multiple baseline measurements, but do not use all of them. Data from a feasibility trial could be explored to investigate MR in, for example, the first 1–2 days of measurement. When comparing multiple trials in a systematic review, the reviewers could consider MR as a source of heterogeneity; for example, investigating trials based on measurement characteristics.

## 4 Conclusions

The present recommendations are designed to raise awareness of how and where taking measurements can lead to bias in trials, so that future studies will have less risk of bias.

We acknowledge that trialists already have a wide range of factors to consider in trial design, and that action taken to address the risk of bias from MR needs to be proportionate and weighed against other priorities and concerns. We hope the practical tools (Table 1 and Fig. 1) provided alongside these recommendations support researchers in making these decisions.

A key finding of the present work is that MR has not been adequately addressed within existing guidelines for designing, reporting (eg, CONSORT) [21] and appraising trials (eg, risk of bias frameworks) [13]. We recommend future iterations of these guidelines refer to our recommendations as a basis to consider where measurement can lead to bias in trials and make revisions where appropriate. For example, it may be useful to consider to what extent the six proposed mechanisms of MR are covered by existing risk of bias frameworks.

Finally, we hope that the many uncertainties identified here act as a stimulus to improved research of this neglected source of bias in trials.

## Figures and Tables

**Table 1:** Trial features that may indicate risk of bias due to measurement reactivity

	Criterion indicating risk of bias	Circumstances under which risk of bias is likely to be higher
**Participant selection**		
	Recruitment	Selection on personal motivation for participation in the trial
	Eligibility criteria	Restrictive eligibility criteria
	Education	More educated e.g. university students
**Measurements**		
	***Features of health outcome of interest***	
	Participant awareness of health-related outcome of interest	Participants aware of outcome of interest (open)
	Nature of health-related outcomes	Outcomes focussed on behaviour or anxiety; health-promoting behaviours such as physical activity
	Social desirability of health outcome	Outcomes with well recognised social norms (e.g. bodyweight)
	***Follow-up***	
	Number of measurement occasions	Measurements repeated on several occasions
	Length of time to follow-up	Short duration likely to be more affected by possible measurement effects
	***Features of measurement procedures/tools***	
	Equivalence of measurement procedures across trial arms	Differential across trial arms
	Similarity between measurement and behaviour change techniques	Measurement directly mimics behaviour change techniques
	Source of data	New data collected specifically for this study
	Measurements open to subjectivity	Self-report measures
	Disclosure of measured values to participants	Values disclosed to participants (immediately)
	Burden of measurement task	Onerous for participant
	Complexity of measurement task	Complex for participant
	Measurement framed in terms of goals/targets	Participants measured against specific goals/targets
	Context	Laboratory setting (as opposed to field or community settings)
**Interventions and comparators**		
	Nature of the intervention	Behavioural and/or self-monitoring components included.
	Blinding to arm allocation	Lack of blinding to arm allocation
**Process evaluation**		
	Process measures	Measures included are assessing mechanisms of action on the primary outcome
	Timing	Conducted before/during trial outcome assessments
	Trial arms included	Conducted only in one trial arm
	Amount of data collected	Extensive data collected from all participants

## Data Availability

The MERIT study did not involve collecting patient or trial data. Further details on the evidence-base used to inform the MERIT recommendations is published elsewhere [6,12]. Any further requests for data submitted to the corresponding author will be considered.

## What is New?

- The MERIT study was funded by MRC-NIHR to produce recommendations about identifying, and ameliorating the effects of measurement on bias in trials of health-related outcomes.
- This is the first set of recommendations on this source of bias.
- Systematic reviews have established that measurement reactivity occurs, but it is not clear under what circumstances it is most likely and of largest effect.
- Fourteen recommendations are presented here to support researchers handle the risk of bias resulting from measurement reactivity by: 1) identifying whether such bias is likely to be a problem for any specific trial; 2) deciding whether to collect further data to inform decisions about whether bias is likely to be a problem for a particular study; and 3) designing trials to minimise the likelihood of bias from measurement reactivity.
- Researchers are urged to use the recommendations presented here to improve their awareness of how and where taking measurements can lead to bias in trials, and to support the design of future trials that will have less risk of bias.

## Box 1 Evidence generated by MERIT study to inform production of current recommendations

We updated an existing systematic review on the question-behavior effect to examine whether asking participants questions had effects on participants’ health-related behaviors. This updated review [6] concluded that these effects were statistically robust across 43 studies,, albeit small in size (standardized mean difference = 0.06, 95% CI 0.02–0.09) and heterogeneous (I^2^ = 54%).

Three rapid systematic reviews were conducted. The first review established that there was no existing guidance on MR. The second review identified existing systematic reviews that quantified the effects of measurement on outcomes, and found five such reviews that have examined the question-behavior effect, with no review of the effects of measurement on affective outcomes (such as feelings and emotions) in health and non-health contexts. The third review identified 16 studies that had examined reactive effects of objective measurements of behavior on health-related behavior, with most evidence of reactivity focused on physical activity and showing small effects and short duration.

A Delphi procedure sought the views of 40 experts to define the scope of the recommendations, in two waves of data collection, and substantial agreement was reached. Finally, a workshop involved discussion of potential recommendations by 23 experts. Recommendations were formed through discussion in groups, with no formal voting procedure to indicate consensus being required.
